# Reproducibility of Volumetric Computed Tomography of Stable Small Pulmonary Nodules with Implications on Estimated Growth Rate and Optimal Scan Interval

**DOI:** 10.1371/journal.pone.0138144

**Published:** 2015-09-17

**Authors:** Gary T. Smith, Ahmad R. Rahman, Ming Li, Brandon Moore, Hester Gietema, Giulia Veronesi, Pierre P. Massion, Ronald C. Walker

**Affiliations:** 1 Department of Medical Imaging, Department of Veterans Affairs, Tennessee Valley Healthcare System, Nashville, Tennessee, United States of America; 2 Center for Quantitative Science, Vanderbilt University Medical Center, Nashville, Tennessee, United States of America; 3 Medisch Spectrum Twente, Enschede, The Netherlands; 4 European Institute of Oncology, Milano, Italy; 5 Section of Pulmonary and Critical Care Medicine, Medical Services, Tennessee Valley Healthcare System, Nashville, Tennessee, United States of America; 6 Departments of Medicine and Cancer Biology, Thoracic Program at the Vanderbilt-Ingram Cancer Center, Nashville, Tennessee, United States of America; 7 Department of Radiology and Radiological Sciences, Vanderbilt University Medical Center, Nashville, Tennessee, United States of America; Stanford University Medical Center, UNITED STATES

## Abstract

**Purpose:**

To use clinically measured reproducibility of volumetric CT (vCT) of lung nodules to estimate error in nodule growth rate in order to determine optimal scan interval for patient follow-up.

**Methods:**

We performed quantitative vCT on 89 stable non-calcified nodules and 49 calcified nodules measuring 3–13 mm diameter in 71 patients who underwent 3–9 repeat vCT studies for clinical evaluation of pulmonary nodules. Calculated volume standard deviation as a function of mean nodule volume was used to compute error in estimated growth rate. This error was then used to determine the optimal patient follow-up scan interval while fixing the false positive rate at 5%.

**Results:**

Linear regression of nodule volume standard deviation versus the mean nodule volume for stable non-calcified nodules yielded a slope of 0.057±0.002 (r^2^ = 0.79, p<0.001). For calcified stable nodules, the regression slope was 0.052±0.005 (r^2^ = 0.65, p = 0.03). Using this with the error propagation formula, the optimal patient follow-up scan interval was calculated to be 81 days, independent of initial nodule volume.

**Conclusions:**

Reproducibility of vCT is excellent, and the standard error is proportional to the mean calculated nodule volume for the range of nodules examined. This relationship constrains statistical certainty of vCT calculated doubling times and results in an optimal scan interval that is independent of the initial nodule volume.

## Introduction

The detection rate of incidental sub-centimeter pulmonary nodules has increased with the advent of thin-cut multislice CT in which the entire lung is visualized with isotropic voxels; however, the clinical significance of these nodules is often uncertain. The Fleischner Society has published guidelines for follow-up of non-screening detected sub-centimeter pulmonary nodules based on patient risk factors and the size of the nodule [1, 2]. Patient risk factor assessment is dependent on accurate clinical history, while measurement of nodule size or volume is dependent on various analytical factors, including the method of measurement. Historically, changes in nodule size have relied on measurement of one or two diameters on the axial view. However, several studies have shown poor reproducibility among radiologists using manual measurement of nodule size [3–5]. Additionally, because lung nodule growth is not limited to the axial plane, sensitivity of these traditional methods to detect a change in nodule size, and particularly a change in volume using subjective visual analysis, is limited.

The advent of high-resolution isotropic CT data with 1.0 or 1.25 mm isotropic voxels offers the ability to perform 3-dimensional estimates of nodule volume [6]. Nodule volume doubling time, *DT*, based on changes in 3-D volume determination, has been used as an imaging biomarker for diagnosis of malignancy[7–10]. Accurate estimate of *DT* depends on reliable determination of nodule volumes for the initial and follow-up scans. These determinations are critically important in an era where we face many more detected nodules [11] and are pressed to decrease the rate of thoracotomy for benign disease [12, 13].

Various authors have investigated the reproducibility of an automated system for determination of nodule volume, so-called volumetric CT, or vCT. However, these studies have utilized either phantoms or same-day (so called “coffee-break”) repeat scans on volunteer patients [14–17]. Neither of these approaches reflects the real world practice of clinical CT in which scans are often performed in varying clinical conditions separated months apart in time. This study addresses those concerns by investigating vCT reproducibility in stable sub-centimeter nodules identified in patients referred for clinical evaluation of indeterminate pulmonary nodules. This paper also focuses on nodules less than ~1,000 mm3 (12 mm diameter), since those nodules are found more frequently as incidental nodules than larger nodules, and larger nodules are typically referred for biopsy for diagnosis.

## Methods

In this paper, nodule growth is assumed to follow a simple exponential growth model with time intervals given in days and nodule volumes given in cubic millimeters. Various authors have defined growth limits of benign and malignant nodules using either the exponential growth rate constant, *k* (day^-1^) or the doubling time, *DT* (days). The doubling time, *DT*, is related to *k* by the simple relation, *DT* = *ln*2/*k*. For our analysis, we used the limits of doubling time, *DT*, for malignant nodules to be between 30 and 400 days. However, we based our mathematical analysis on the exponential growth rate constant, *k* (day^-1^), since it is normally distributed around zero for stable nodules, while *DT* is not.

### vCT Patient Studies

The Tennessee Valley VA Healthcare System Institutional Review Board approved this retrospective study, and waived the requirement for subject informed consent. Nodule volume determination was performed on archived CT images on the TVHS PACS system by a single investigator (GTS). All data was then transferred to a de-identified database for statistical analysis. We retrospectively reviewed vCT scans in 71 consecutive patients (64 male, 7 female), aged 38–79 yrs (mean 62 yrs), who underwent from three to nine repeat vCT scans over three years for clinical follow-up of known indeterminate pulmonary nodules between 12/16/2009 and 7/1/2012. In these patients, we identified 213 non-calcified nodules. Of these, 89 were found to be less than 1300 mm^3^ and deemed stable by visual and caliper measurements as well as showing no more than 25% deviation in estimated volume by vCT using a commercial lung nodule software analysis package (VCAR™, GE Medical Systems, Waukesha, WI). Because of limitations of the VCAR™ vCT software to accurately extract sub-solid, non-solid, partially calcified, subpleural and perivascular nodules, these types of nodules were excluded from this analysis. In order to validate our results in non-calcified nodules, we applied the same volume determination algorithm and statistical analysis of 49 densely calcified pulmonary nodules in the same patient population. Densely calcified nodules due to prior granulomatous disease are common in our patient population and are typically stable for many years.

All scans were performed for clinical interpretation using a GE Discovery 64-slice CT (GE Healthcare, Waukesha, WI) with dose optimization software at 120 kVp with variable mAs, 0.8–1 sec gantry rotation, and contiguous 1.25 mm slice thickness. To better approximate potential clinical practice, no correction was made to the nodule volume determination for variable scan kVp and mAs. Image reconstruction was by filtered back-projection. Each CT scanned averaged 1.5 mSv effective dose. All subjects were at high risk for lung cancer as defined in the National Lung Screening Trial[18].

**Fig 1:**
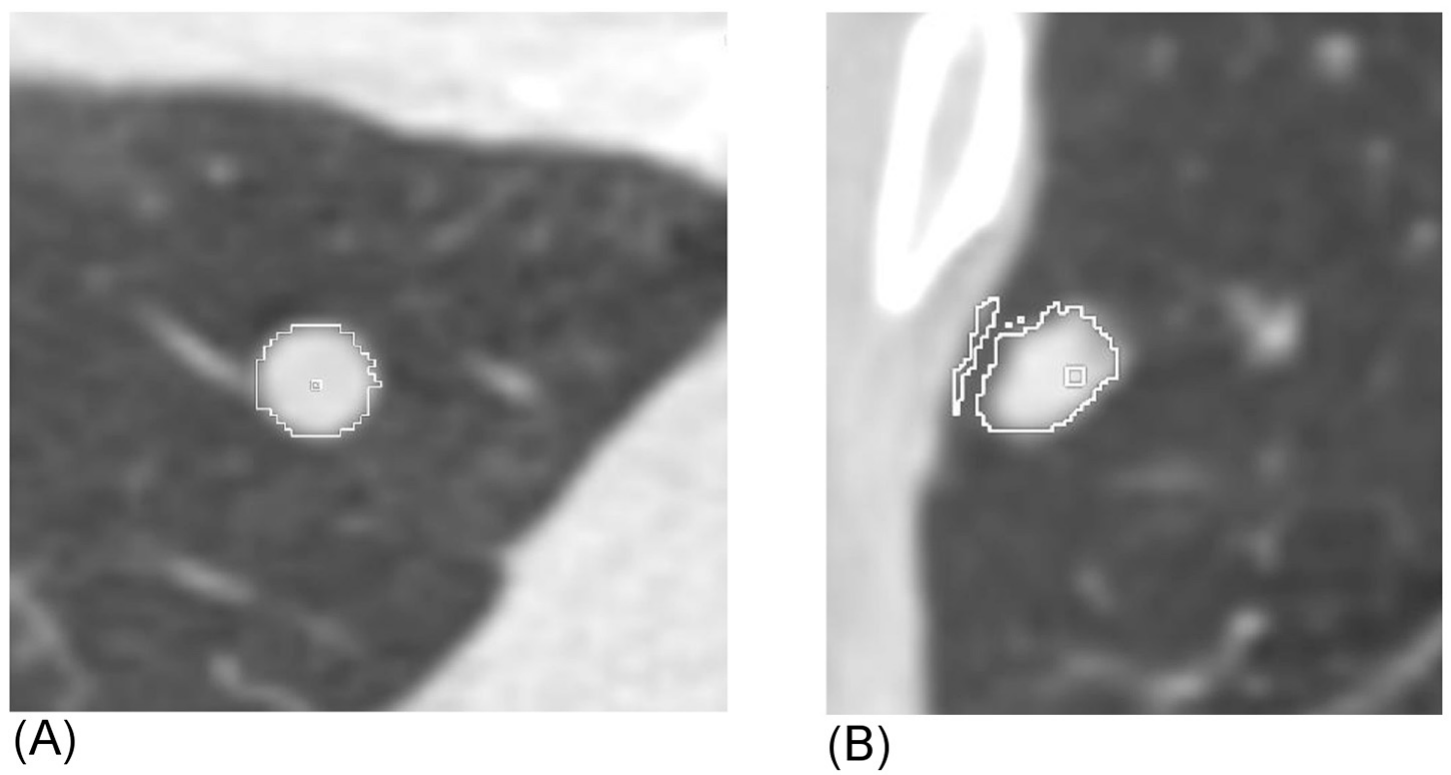
Example of automated volume extraction of a calcified nodule by VCAR software. **(A)** Successful extraction: Note the contour fit to the nodule perimeter in multiple projections appears to correlate with the visual nodule outline. **(B)** Unsuccessful extraction: Note the clear erroneous border that includes adjacent chest wall in this subpleural nodule.

Nodules were identified by visual inspection of the scan and marked with a software fiducial “seed” using VCAR™ vCT software by a physician experienced in the interpretation of chest CT examinations. The 3D contours determined by the software were visually analyzed for accuracy, and those that were clearly inaccurate, even after attempting to manually adjust the processing volume (typically due to inclusion of an adjacent vessel or nearby pleura) were excluded from analysis (Fig 1). On repeat scans, all nodules were re-identified and matched to their corresponding nodules on the new study. VCAR™ volume determination was performed on CT images reconstructed with a standard soft tissue kernel as recommended by the VCAR^TM^ users’ manual. All volumes were recorded along with the dates of the scans.

### Analytical plan and statistical considerations

Given the assumption that nodules determined to be stable by visual and caliper analysis, as well as falling within the 25% difference threshold, are truly stable, any change in calculated volume was attributed to measurement variation rather than physiologic nodule growth or regression. Because more than two measurements were made of all nodules, Bland-Altman analysis could not be used to determine reproducibility. Instead, as recommended by Bland and Altman for repeated measures[19], we used linear regression to determine the relationship of the nodule volume standard deviation, *σ*_*V*_, to the average nodule volume, $\bar{V}$, according to the equation $\sigma_{V}\;=\;a\overset{-}{V}$, where *a* is the slope of the best-fit linear least squares regression line.

### Computation of False Positive and True Positive Rates

**Fig 2:**
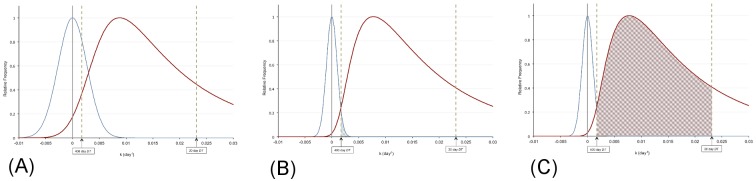
Normalized population distributions for stable and malignant nodules as a function of the scan interval, *ΔT*. The stable nodules are described by a normal distribution centered at *k* = 0 zero and the malignant nodules are described by a lognormal distribution. **(A)** 30-day scan interval. **(B and C)** 81-day scan interval. Note the decrease in width of the distributions with a longer scan interval due to the effect of the scan interval time, *ΔT*, on the standard deviation for *k* as given by Eq 4 in the Appendix. In Fig 2B, *FPR* is computed as the percent area under the stable curve between the 400-day and 30-day doubling time values (0.00173<*k*<0.0231 day^-1^) (vertical dashed lines). In Fig 2C, *TPR* is computed as the percent area under the malignant curve between the same limits.

The mathematical basis for calculating the false positive rate (*FPR*) and true positive rate (*TPR*) is described in the Appendix. Graphically, this process is illustrated in Fig 2, which shows the distribution of the growth rate, *k*, for both stable nodules and malignant nodules at 30 and 81 day scan intervals, *ΔT*. (Note: In Fig 2, the 30 day scan interval was chosen to amplify the effect of a short *ΔT* on the shape of the curves for visual clarity. The 81 day interval was chosen for comparison since that is the optimal *ΔT* determined by this study, as described in the Results section.) The curves in Fig 2A are derived from a normal distribution about *k* = 0 for stable (benign) nodules, and a lognormal distribution of growing (malignant) nodules, each using the standard error given by Eq 4 in the Appendix. The *FPR* can be seen in Fig 2B to be the area under the stable nodule distribution curve between the limits of 30 to 400 days, *k* = ln2/400 to *k* = ln2/30 day^-1^ (i.e. 0.00173<*k*<0.0231 day^-1^). The *TPR* is shown in Fig 2C as the area under the malignant nodule distribution curve between the same limits. As can be seen by comparing the distributions, the *FPR* and *TPR* distribution widths are narrower for a larger *ΔT*, thus enabling determination of an optimal scan interval to ensure *FPR*<0.05.

To determine the actual *FPR* of our data, we computed the growth rate constant *k* for all stable nodule pairs using all forward-in-time scan intervals. For example, for a nodule with 4 scans, *k* was calculated using scan pairs 1–2, 1–3, 1–4, 2–3, 2–4, and 3–4. The *FPR* was determined by counting the percentage of occurrences in which the *k*-value fell into the malignant limits corresponding to doubling times between 30 and 400 days (i.e. 0.00173<*k*<0.0231 day^-1^). To validate this method, we also computed the *FPR* from Gietema’s[15] “coffee break” study of 217 nodules scanned twice in a single day using simulated scan intervals of 30–180 days.

The area under the normalized lognormal malignant curve in Fig 2C between the *k*-value limits corresponding to 30–400 day doubling times yields the *TPR*. Because the error in the determination of *k* for a malignant nodule is dependent on the scan interval as given by Eq 4 in the Appendix, the shape of the malignant distribution curve is dependent on the scan time interval, *ΔT*. That is, *ΔT* acts as a “blurring function” on the distribution of malignant nodules, with short *ΔT* increasing the width of the malignant distribution curve. *TPR* then given by the area under the “blurred” malignant distribution curve as described in the Appendix. For this analysis, we converted the distribution of malignant doubling times published by Veronesi [9] to a lognormal distribution of growth rates, *k*.

## Results

Patients were followed for an average of 1.9 yrs (range 0.6–4.2 yrs). Of the 89 nodules, 44 had three scans, 26 had four scans, 9 had five scans, 6 had six scans, 1 had seven scans, and 3 had eight scans. This provided a total of 583 two-scan data pairs for calculation of *FPR*. Of the 42 patients, 22 had one nodule, 12 had two nodules, 6 had three nodules, two had 4 nodules, two had 5 nodules, and one patient had 8 nodules.

**Fig 3:**
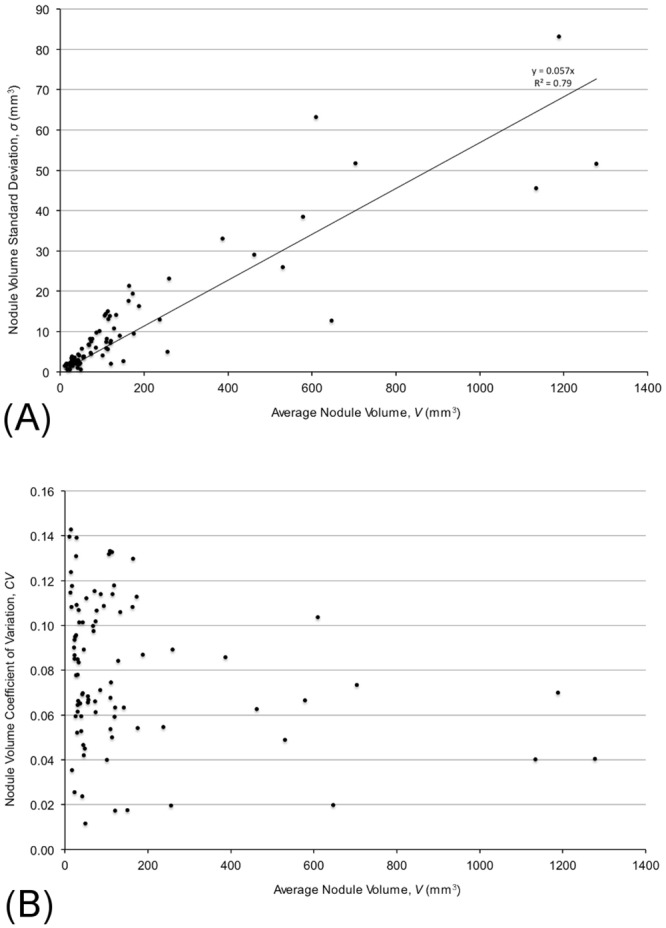
Error in nodule volume determination. **(A)** Plot of nodule volume standard deviation *σ*_*V*_ versus mean nodule volume $\overset{-}{\;V}$ (mm^3^) for all datasets. Linear regression for our non-calcified nodules (solid line) gives, $\sigma_{V}\;=\;0.057\overset{-}{V}$ (*a* = 0.057 ± 0.002), r^2^ = 0.860, p = <0.001). The dotted line shows the same analysis applied to Gietema single-repeat data $\sigma_{V}\;=\;0.054\overset{-}{V}$ (*a* = 0.054 ± 0.003), r^2^ = 0.590, p = <0.001). **(B)** Coefficient of Variation for all nodules. Above 100 mm^3^, CV is below 0.15.

Fig 3A shows a plot of nodule volume standard deviation, *σ*_*V*_, versus the mean nodule volume, $\overset{-}{\;V}$, for all 89 stable nodules. Linear regression (solid line) yielded $\sigma_{V}\;=\;0.057\overset{-}{V}$ (*a* = 0.057 ± 0.002; r^2^ = 0.79, p = <0.001). This is not significantly different from the same analysis applied to the single repeat “coffee break” data of Gietema (dashed line in Fig 3A). The coefficient of variation, *C*_*ν*_, defined as the standard deviation divided by the mean, is less 0.15 for all nodules (Fig 3B).

In our review of stable calcified nodules, we detected 49 calcified nodules between 4–10 mm diameter that were successfully segmented using the VCAR^TM^ software. The standard error as a function of the mean volume for these nodules was $\sigma_{V}\;=\;0.052\overset{-}{V}$ (slope = 0.052±0.005; r^2^ = 0.65; p = 0.03).

**Fig 4:**
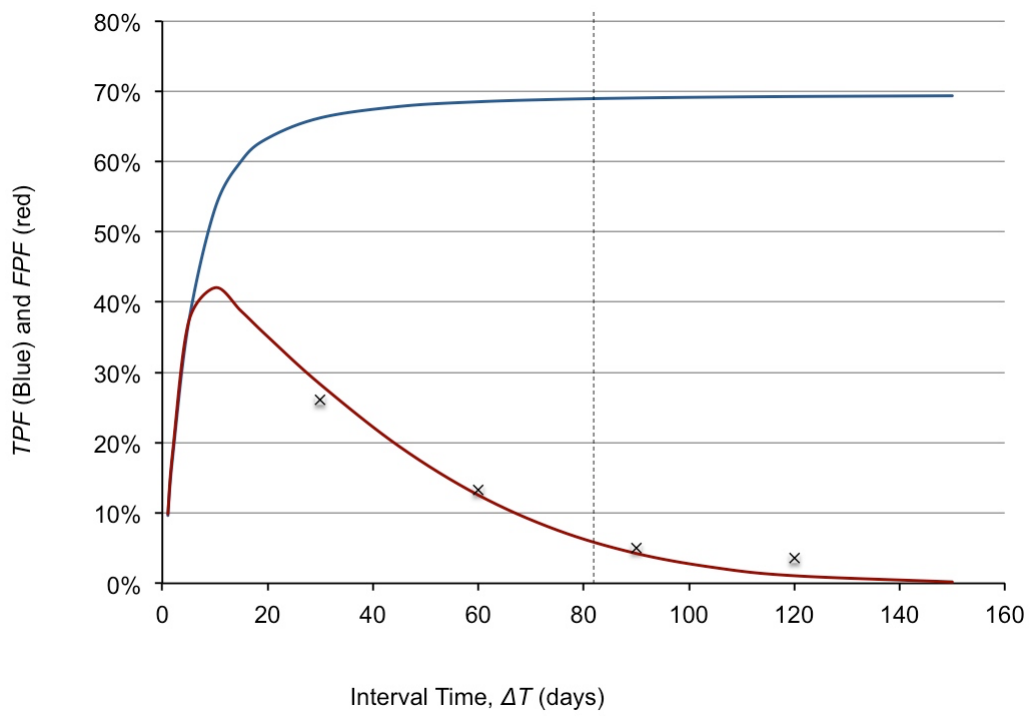
Calculation of True Positive Rate (TPR) and False Positive Rate (FPR). After 81 days (dashed line), the *FPR* is <5%, with a *TPR* at a maximum value of 72% as determined by the *k*-distribution in 120 malignancies[9]. This relationship is independent of nodule volume due to the linearity of the standard error shown in Fig 3. Our calculated *FPR* agrees well with values computed from Gietema’s[15] “coffee break” study (“x” labels shown on the graph) with simulated scan intervals of 30–180 days.

Computation of the false positive rate, *FPR*, and the true positive rate, *TPR*, as a function of the scan time interval, *ΔT*, according to the methods described in the Appendix yielded the results shown in Fig 4.

**Fig 5:**
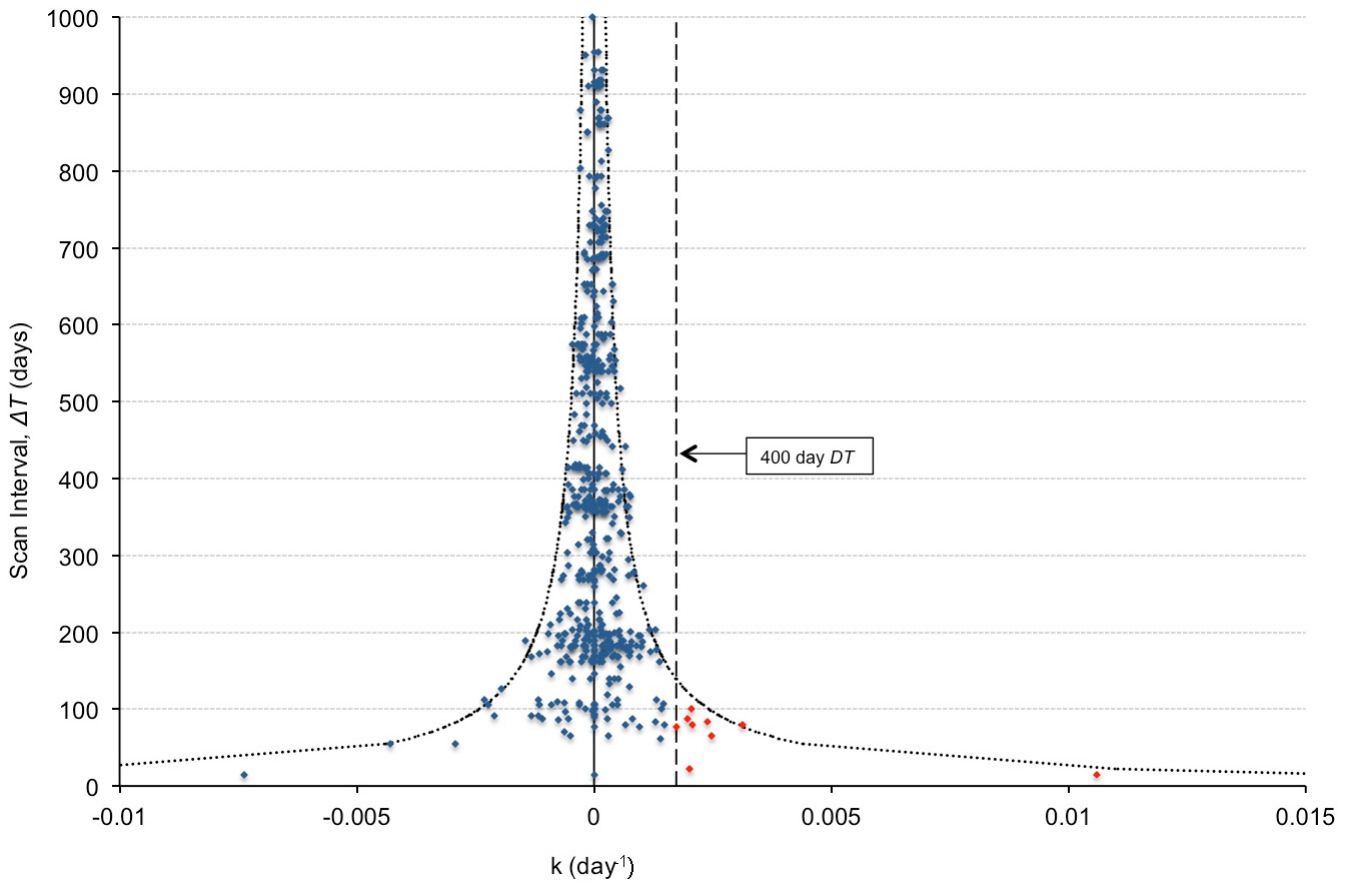
Plot of stable nodule *k* (growth rate) estimates for all non-calcified nodules as a function of scan interval, *ΔT*. The estimated *FPR* is 1.6% (9/579), as computed by *k* > 0.00173 day^-1^ (i.e. *DT*<400 days) (horizontal dashed line). There were no false positive results for a scan interval greater than 100 days. Note the decreasing error in *k* with increasing scan interval time. The border curves (•••••) represent ±3*σ*_*k*_, as determined from Eq 4 in the Appendix, with *a* = 0.057 as calculated in Fig 3A.

As expected, *FPR* decreases with increasing *ΔT*, and *TPR* increases to an expected maximum value determined by the pure lognormal fit without “blurring”. Note that after 81 days, *FPR* was less than 5% with the maximum *TPR* of 72%. Using our paired stable nodule data, the calculated *FPR* was 1.6%, with a maximum scan interval of 100 days (Fig 5). Using Gietema’s “coffee break” data, the *FPR* was 5% or less at a scan interval of greater than 90 days.

## Discussion

We report our experience using VCAR™ software to determine reproducibility of volumetric CT in assessing stable non-calcified indeterminate nodules. Unlike prior studies on reproducibility of volumetric CT using phantoms or same-day follow-up scans of patients, this study demonstrates the reproducibility of quantitative vCT in a “real-world” clinical setting with follow-up examinations spanning over three years, and in particular for small indeterminate pulmonary nodules.

In this study, we excluded nodules that we could not segment with VCAR^TM^ software. This represents approximately 30% of nodules, with the failure to segment typically due to adjacent structures including the chest wall, vessels, and fissures. This is consistent with other studies showing up to 51% inappropriate segmentation [15]. For the 89 stable non-calcified nodules with acceptable extraction by VCAR™ (determined by visual inspection of the contours), the standard deviation of the nodule volume was linearly related to the mean volume (Fig 3A). This finding was unexpected since we initially believed that the system variance would be constant for all nodule volumes or would decrease with increasing nodule volumes [17, 20]. Our model reflects the findings of Gietema [15] as demonstrated in her Bland-Altman analysis showing increase in nodule size difference with increased mean volume, as well as by Xie, et al, (21) who have shown variations of the standard Bland-Altman plots using percent difference rather than absolute difference.

This study assumes that all of our subjects’ non-calcified nodules were indeed stable over the course of the study. In fact all these nodules had a minimum follow-up time greater than 24 months. In order to validate this presumption, we undertook a selected study on incidentally detected calcified nodules in our patient population and found similar results, suggesting that results on the non-calcified dataset are accurate.

Using the distribution of doubling times published by Versoni [9], suggests that approximately 28% of malignant nodules will be misclassified (*TPR* = 72%) regardless of either the initial nodule volume or the scan interval. This is slightly higher than the false negative rate by CT reported in the first phase of the NELSON trial of 22% [8], but agrees with the 27% false negative rate reported by Wang[21]. One reason our results may be higher than the NELSON trial is that it is likely that Veronesi’s data is “blurred” by the same process as described for the *TPR* calculation in the Appendix, and therefore de-convolution may give a closer estimate to that seen in the NELSON trial.

It is important to note that the *FPR* of benign nodules is also dependent on the follow-up scan interval but not the nodule volume. Because of statistical variation in the volume determination, it is possible to estimate a stable nodule as smaller than the true value on the first scan and then larger on the second scan, giving the illusion of nodule growth. If the scan interval is short, the growth rate might be calculated to be rapid enough to falsely classify it as a malignant nodule, while a longer scan interval is less likely to do so. This is easily seen by rewriting the exponential growth model estimate for the growth rate as, *k* = Δ(*lnV*)/Δ*T*, where a short time interval can lead to large values of *k*. For our stable nodule paired data, *FPR* was estimated to be 1.6%, all with follow-up intervals of 100 days or less. Our *FPR* of 1.6% at *ΔT* = 100 days is less than that predicted by the process outlined in the Appendix. This is not unexpected, however, given the fact that we used nodules with stability within 25% as done in the NELSON trial, but not included in our mathematical model.

It is important to note from Eq 4 in the Appendix that variation in the calculated growth rate due to statistical error in each vCT volume measurement is independent of nodule volume. This means that misclassification of malignant nodules is also independent of nodule size, and that optimal follow-up interval to reduce false negative misclassification errors when using VCAR^TM^ software should not be dependent on nodule size. In our stable non-calcified nodule data, we had 9 false positive misclassifications: one sub-4 mm nodule, five 4–5.9 mm nodules, and three 6–7.9 mm nodules. There were no false positive misclassifications for >8 mm nodules. Realizing that other authors [15, 22] have shown Bland-Altman data with increasing variance with increasing nodule size suggests that using nodule size to direct follow-up scan intervals may be unnecessary for those vCT systems as well.

This study has limitations. One limitation of this study is the determination of stability in non-calcified nodules. To address this, we also measured a group of 49 calcified nodules in the same patient population using the same VCAR^TM^ software. Our results in calcified nodules support the results from our stable non-calcified nodules, suggesting that future work might be able to be done using calcified nodules as a “calibration standard” for testing system reproducibility.

Interestingly, we did not see decreasing error in nodule volume determination with increasing nodule size as described by Kostis, et al [23], when using non-calcified nodules determined to be stable by repeat scans up to two years. This may be a function of differences in the segmentation algorithm. Segmenting nodules for volume determination is software dependent, and other software may result in different sources of error. However, data from Gietema and Xie [15, 22] both show similar results to our study, each using different software.

Another limitation of this study is the use of a soft tissue reconstruction kernel rather than a lung kernel. This was done because of the difficulty visually separating sub-centimeter non-calcified nodules from calcified nodules when using a high frequency reconstruction filter. It is uncertain what the effect of using a lung kernel would have on the variance due to potential offsetting effects of sharper edge definition and increase in noise. Further work to determine the optimal reconstruction kernel for vCT remains to be done. This is further complicated by the recent introduction of iterative reconstruction algorithms for CT. Accordingly, changes in nodule density and/or volume (the two parameters objectively quantified with VCAR™ or similar software) may be amenable to reproducibility only if each CT examination is performed on the same instrument with rigidly controlled calibrations and techniques, though this has not yet been adequately studied.

Also, while nodule volume and density measurements are easily automated for completely intraparenchymal nodules, VCAR™ software was not reliable for pleural-based nodules or perivascular nodules, so such nodules were excluded from this analysis. Whether these nodules could be analyzed by interactive involvement with the software by the radiologist (e.g. adjusting nodule contours visually) or improvements in software design remains to be determined.

In conclusion, this study shows the reproducibility of the VCAR™ software automated nodule volume determination when used with a consistent CT scanning protocol, and its potential effect on the accuracy of vCT for detecting nodule growth in clinical follow-up. Our results show excellent reproducibility of VCAR^TM^ software in a “real world” clinical setting. There was a linear relationship between the standard error in volume determination and estimated nodule volume, with larger nodules showing larger error in vCT volume determination compared with smaller nodules. Use of this relationship to predict false positive and false negative misclassification fractions agrees well with published data, and may enable better recommendations for interval follow-up of indeterminate pulmonary nodules when using vCT.

## Appendix

### Mathematical Basis for Calculation of False Positive Rate and True Positive Rate

Nodule growth is assumed to follow a simple exponential growth model,

$$V(t)=V(0)e^{kt}$$ (1)

where *k* is the exponential growth rate constant (1/days) and *t* is given in days. For a nodule measured at time points, 1 and 2, separated by a known time interval, Δ*t*, solving for *k* yields

$$k=\frac{1}{\Delta t}ln\left(\frac{V_{2}}{V_{1}}\right)$$ (2)

If the variance in the measured volumes, *V*_*1*_ *and V*_*2*_, is known, then the variance in the exponential growth rate, $\sigma_{k}^{2}$, can be estimated using the error propagation formula,

$$\sigma_{k}^{2}={\left(\frac{\partial k}{\partial V_{1}}\right)}^{2}\sigma_{V_{1}}^{2}+{\left(\frac{\partial k}{\partial V_{2}}\right)}^{2}\sigma_{V_{2}}^{2}$$ (3)

If the volume standard deviation is best described as a linear function, *σ*_*V*_ = *aV*, then Eq 3 yields an expression for the growth rate standard error given by

$$\sigma_{k}=\frac{a\sqrt{2}}{\Delta T}$$ (4)

Note that *σ*_*k*_ is independent of the nodule volume, *V*.

### Computation of False Positive Rate

Assuming all stable nodules have zero growth, the false positive rate, *FPR*, becomes the normal distribution function with mean of zero and standard deviation given by Eq 4 integrated between the limits, *k* = ln2/400 to *k* = ln2/30 day^-1^ (using doubling times from 30 to 400 days as positive for malignancy). We used the Excel (Microsoft, Inc., Renton, WA) cumulative distribution function, norm.dist(x,*μ* = 0,*σ* = $a\sqrt{2}/\Delta T$, 1), for x = 0.0231 (*DT* = 30 days) and x = 0.00173 (*DT* = 400 days), to compute the value for *FPR* at various time intervals, Δ*T*, using an experimentally determined value of *a* as shown in Fig 3A. Using this expression, it is possible to select the optimal scan interval for any clinically desired false positive rate.

### Computation of True Positive Rate

**Fig 6:**
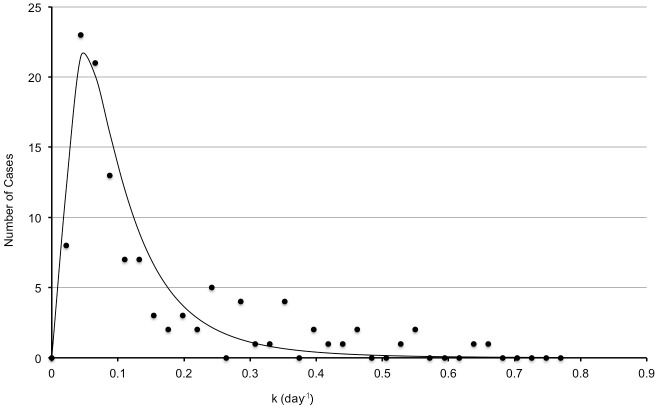
Malignant nodule growth rate distribution. Distribution of *k*-values for 120 malignant nodules as determined by Veronesi[9]. The dashed line represents the best fit lognormal distribution with mean ln(0.015) and standard deviation 0.83.

If the true distribution of malignant nodule growth rate is known, then the true positive rate, *TPR*, i.e., the percent of malignant nodules have a calculated doubling time of between 30 and 400 days (i.e. 0.00173<*k*<0.0231 day^-1^) can be computed. We obtained this distribution from published data given by Veronesi[9] of doubling times of malignant nodules taken at long interval times. First, the Veronesi data was transformed to plot a histogram of the number of malignant cases versus *k* instead of doubling time. The resulting curve was fit to a lognormal distribution with mean, *μ*_*L*_, and standard error, *σ*_*L*_. Values for *μ*_*L*_ and *σ*_*L*_ were found using a least squares algorithm to be ln(0.015) and 0.83, respectively (Fig 6).

Since the variance in *k* acts as a “blurring” function for the histogram given in Fig 6 at each scan interval, *ΔT*, the estimated number of malignant cases, *N*, at any scan interval is determined by the convolution of the malignant nodule lognormal distribution with a normal standard error distribution with $\sigma_{k}\;=\;a\sqrt{2}/\Delta T$, as given above. *TPR* is then given by the integral of *N* between 0.00173<*k*<0.0231 day^-1^. We computed this numerically for different values of *ΔT*.
